# Food Consumption Behavior of Pakistani Students Living in China: The Role of Food Safety and Health Consciousness in the Wake of Coronavirus Disease 2019 Pandemic

**DOI:** 10.3389/fpsyg.2021.673771

**Published:** 2021-07-27

**Authors:** Muhammad Khayyam, Shuai Chuanmin, Haroon Qasim, Muhammad Ihtisham, Raheel Anjum, Li Jiaxin, Anna Tikhomirova, Nawab Khan

**Affiliations:** ^1^School of Economics and Management, China University of Geosciences, Wuhan, China; ^2^School of Business and Management Sciences, Minhaj University, Lahore, Pakistan; ^3^College of Horticulture and Forestry, Huazhong Agricultural University, Wuhan, China; ^4^College of Landscape Architecture, Sichuan Agricultural University, Chengdu, China; ^5^Department of Economics, Abdul Wali Khan University, Mardan, Pakistan; ^6^College of Management, Sichuan Agricultural University, Chengdu, China

**Keywords:** COVID-19, sojourners in China, food safety, health consciousness, Theory of Planned Behavior, PLS-SEM

## Abstract

The emergence of coronavirus disease 2019 (COVID-19) has considerably changed global food production, processing, and consumption at different levels. Sojourners are among those who have experienced a higher level of food insecurity during the crisis of the COVID-19 outbreak. The current research aimed to investigate the immediate consumption behavioral intentions of the Pakistani international students in the People**'**s Republic of China (PRC) during the wake of COVID-19 pandemic. This study applied the Theory of Planned Behavior (TPB) and background factors of food safety and health consciousness that influence the consumption behavioral intention of Pakistani students toward unfamiliar local food in China. A relational model was analyzed where food safety and health consciousness were hypothesized to serve as background variables associated with TPB components. Moreover, the indirect effects of food safety and health consciousness on behavioral intentions were assessed. The data were collected through convenience samples from 462 Pakistani international students and were analyzed through partial least square structural equation modeling (PLS-SEM). The results confirmed that food safety and health consciousness were positively associated with attitude (ATT), subjective norm (SN), and perceived behavioral control (PBC). However, food safety and health consciousness were indirectly associated with the behavioral intention only through ATT and SN. The results highlighted the role of food safety and health consciousness as important antecedents of classical TPB components that affect intentions and behaviors to avoid unfamiliar local food in a migrated context. The present study provides enlightenment to those who aim to investigate the consumption behavioral intentions of sojourners in the wake of the pandemic situation based on food safety and health consciousness. The findings of the current study are also applicable to general consumption patterns in the food sector.

## Introduction

Coronavirus disease 2019 (COVID-19) is an infectious disease caused by the novel coronavirus, severe acute respiratory syndrome coronavirus-2 (SARS-CoV-2). It is a highly contagious disease identified in late December 2019 and declared as a global pandemic by WHO on 11 March 2020 (COVID-19 Dashboard, 2020). Initially identified in the city of Wuhan (Hubei, China), the ongoing COVID-19 has spread globally, and as of 21 February 2021, caused 110,749,023 confirmed cases and 2,455,131 deaths worldwide (COVID-19 Dashboard, 2021). Based on some evidence (not the firm), the first infections of the disease were linked to Hunan Seafood Market, located in Wuhan city (Laborde et al., 2020). The sequencing technology of genes showed that the SARS-COV-2 poses a similarity in gene sequence with the bat coronavirus up to 96.2%, thus suggesting bats as the possible source of the disease (Zhou et al., 2020).

The emergence of COVID-19 has considerably changed global food production, processing, and consumption at different levels. The concerns for individual hygiene and consumer behavior have been significantly shifted with the prevalence of COVID-19 (Olaimat et al., 2020). The increasing health and food safety concerns have turned the behavior of the consumers toward a healthier and more sustainable direction, resulting in the avoidance of unfamiliar food products (Butu et al., 2020). For instance, the results of Qi et al. (2020) revealed that the COVID-19 crisis had influenced the perceptions and attitudes of consumers toward green foods. Shreds of evidence suggest that the ongoing pandemic crisis has a considerable influence on the life and consumption behavior of the sojourners (Liyanage, 2020; Morales et al., 2020). However, none of the studies have systematically investigated the food consumption behavior of the sojourners in the context of the COVID-19 pandemic. Sojourners are among those who have experienced a higher level of food insecurity during the coronavirus outbreak. Recently, researchers have attempted to explore the food patterns of the sojourners, which provide a comprehensive description of motivational factors of the food preferences of the sojourners during their sojourning (Brown et al., 2010; Wu et al., 2016; Xuhui et al., 2018; Yu et al., 2019). Research attention toward food consumption of the sojourners during COVID-19 is yet limited, rendering it a challenge for the destination marketers, tourism and hospitality businesses, and policymakers. The current research contributes to the scarce literature on the food preferences of the sojourners during the COVID-19 pandemic, concerning food safety and health consciousness. This research significantly aims to investigate the consumption behavioral intention of Pakistani students living in People's Republic of China (PRC) during the COVID-19 pandemic.

The PRC has become a hot corner for an increasing number of expatriates and is ranked as a highly attractive destination for foreigners in 2017 (HSBC Holdings plc, 2017). Besides, the Chinese Ministry of Education has enrolled enormous academic sojourners, which created a reputation as a regional center for education (Global Times, 2020). In 2019, there were 492,185 international students from 196 countries studying in PRC, where Pakistan was ranked as the second-highest number of international students (TRIBUNE, 2019). The strong bilateral relations between the two countries and China Pakistan Economic Corridor (CPEC), has set China as the top education destination for Pakistani international students. With the increasing number of Pakistani academic sojourners and their contribution to the economic and education sector of China, it is crucial to clearly understand the challenges they face during their stay in PRC. Their move to the new Chinese cultural environment with key communication barriers, cultural and social disparities, economic imbalance, and consumption differences represent the most traumatic situation they can experience. In this situation, some level of food shock is unavoidable. To the best of our knowledge, none has attempted to investigate the consumption behavior of Pakistani students living in China, particularly during the COVID-19 pandemic, leaving a knowledge gap to understand this potential market segment.

The traditional Pakistani culture is food-centered (Usman et al., 2020). Pakistani foods form part of the collectivistic cultural and religious identity, where group membership is essential (Sadia et al., 2021). Given its religious and robust cultural importance to Pakistani food, it is not surprising that Pakistani consumers have stronger ethnic and food retention when traveling abroad (Kamran, 2021). Mirza (2020) investigated that the second and even third-generation Pakistani immigrants living in the UK, daily consume Pakistani food and prefer to choose Pakistani restaurants when eating outside. Despite being strongly attached to the traditional Pakistani food, most young Pakistani consumers prefer to consume global food brands on several occasions (Sadia et al., 2021). However, due to the scant research on Pakistani students living in China, it remains uncertain if they are inclined to show similar interest in the local Chinese or global food brands after their initial arrival in China. Besides, with the emergence of COVID-19, consumers today are more resistant to consuming unfamiliar food, specifically in a migrated or expatriated context where they have less information regarding the safety of local foods (Morales et al., 2020). The current situation leads to increased health and safety concerns that negatively affect local or unfamiliar food consumption (Leone et al., 2020; Olaimat et al., 2020; Qi et al., 2020). Therefore, due to the increasing number of Pakistani students, food uncertainties, and recent scandals in the Chinese food market, diverted the intentions of the authors to conduct contemporary research that is more predictable and pertinent to the new circumstances.

## Theoretical Background

During the last decades, the food choices of consumers have been thoroughly investigated. The literature has acknowledged several socio-psychological theories which act as foundations to investigate food consumption patterns. Based on the intricacy of food choices, this research approaches to employ the Theory of Planned Behavior (TPB) as a theoretical basis to investigate the intended consumption behavior. The TPB emphasizes on precise interested behavior which offers a complete model that explains/understands the factors determining the interested behavior. The TPB proposes that specific behavior (BEH) is evaluated through the intention to perform it (Ajzen, 2002). The intention is the direct precursor of the actual behavior, which captures motivations and cognitive planning. The intention is further determined by three main variables, such as personal attitude (ATT), subjective or social norm (SN), and perceived behavioral control (PBC) (Ajzen, 2012). Over the decades, the TPB has played a vital research role in various disciplines. Many studies have demonstrated the relationship of the prediction power of TPB with the consumption behavior of individuals (Bonne et al., 2007; Singh and Verma, 2017; Soon and Wallace, 2017; Ting et al., 2017; Ali et al., 2018; Giampietri et al., 2018; Mohd Suki and Abang Salleh, 2018; Canova et al., 2020) that justify its application in the current research.

## Aims and Hypothesis

Subsequent to TPB, firstly, the current research aims to predict and explain the consumption behavioral intentions of young Pakistani international students to retain Pakistani food in a migrated context, particularly during the crisis of COVID-19. Secondly, the study aims to investigate how concerns about food safety influence the behavioral intentions of Pakistani students to avoid unfamiliar or local food during their sojourning during the period of COVID-19. Thirdly, based on the prime reason for health concerns, this study aims to investigate the influence of health consciousness on the behavioral intentions of Pakistani students to avoid unfamiliar food products.

Attitude refers to the psychological tendency that describes the self-performance evaluation of an individual, which predicts intentions and, consequently, the actual behaviors. It implies the degree of positive or negative evaluations toward a behavior (Ajzen, 2002). Numerous studies have reported the positive associations between attitude and intentions to perform the behavior of interest (Azam, 2016; Singh and Verma, 2017; Xu et al., 2020). Similarly, several studies have reported significant positive relationships between intentions and attitude toward consuming ethnic food (Leung, 2010; Arvela, 2013; Ayyub, 2015; Ting et al., 2019). Chang et al. (2010) investigated that Chinese tourists have a positive attitude toward consuming Chinese food when traveling abroad. Based on the previous results, we argue that local Chinese food can be attractive to Pakistani students, but their unfamiliarity is also an obstacle for consumption; therefore, we hypothesize that:

H1. Attitude has a significant positive effect on the intentions of Pakistani students to avoid unfamiliar local food and retain Pakistani food consumption during their sojourning in China, particularly during the period of COVID-19.

Apart from the attitudinal effect, social influence also plays a vital role in the specific food consumption behavior. The said influence is termed as SN in TPB. It reflects the perceived external pressure on individuals either to perform the behavior or not to perform the behavior (Ajzen, 2011). Park (2000) defined SN as the sentiment of the individuals with regard to the social pressure received from important referents. Mohd Suki and Abang Salleh (2018) argued that the social/religious groups form the foundations of community life, eventually leading them to grasp strong social values with other individuals living in the same communities. In this case, those who highly consider themselves as a part of the social group will be highly motivated to comply with the group members (Ali et al., 2018). Wu et al. (2016) investigated that the social pressure on the Chinese tourists to avoid unfamiliar local food negatively affects their intention to consume unfamiliar food. Consequently, we hypothesize that:

H2. Subjective norm has a significant positive effect on the intentions of Pakistani students to avoid unfamiliar local food and retain Pakistani food consumption during their sojourning in China, particularly during the period of COVID-19.

Behavioral control means “the perceived easiness/difficulty to conduct the behavior of interest” (Ajzen, 2002). The PBC predicts both intention and actual behavior; however, the strength of the relationship between the behavioral control and intention differs across studies (Canova et al., 2020). In many cases, PBC has a substantial positive effect on the intention to consume a specific kind of food (Moser, 2016; Carfora et al., 2019; Lim and An, 2021); whereas, in some cases, the relationship was insignificant (Ayyub, 2015; Wibowo and Ahmad, 2016; Ali et al., 2018). Similar deliberations were found in the context of several ethnic foods (Bonne et al., 2007; Ting et al., 2016, 2019). The conflicting arguments of researchers may be associated with the availability of the investigated food for a specific group (Mohd Suki and Abang Salleh, 2018) which helps them to grasp control over the desired behavior. Conversely, individuals who fail to grasp control will not be able to perform the behavior. Based on the above discussion, we hypothesize as follows:

H3. PBC has a significant positive effect on the intention of Pakistani students to avoid unfamiliar local food and retain Pakistani food consumption during their sojourning in China, particularly during the period of COVID-19.

**Figure 1:**
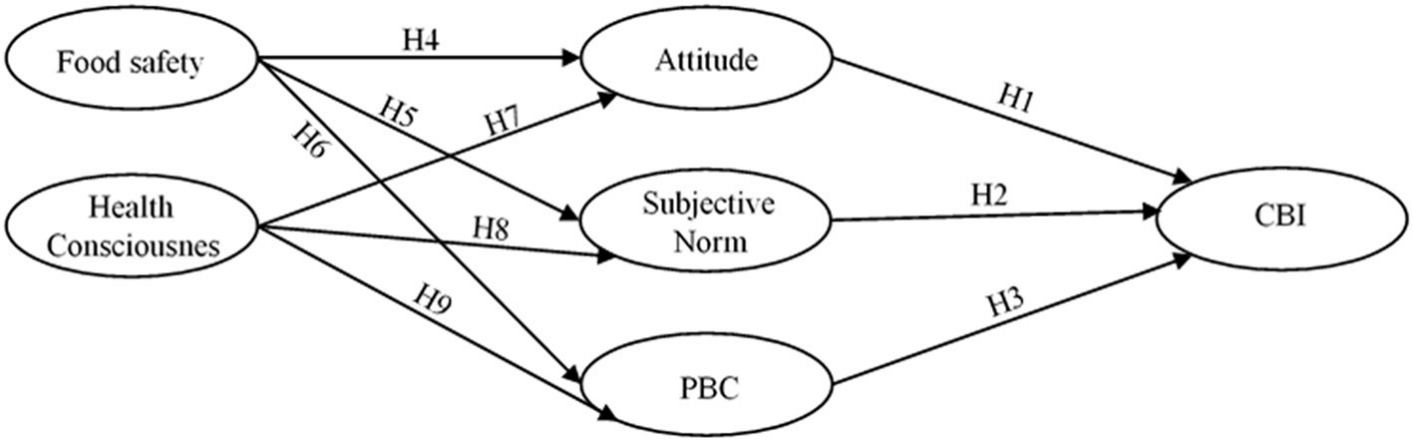
Hypothesized model.

Suggested by academic literature, TPB allows some background variables (e.g., self-identity, risk perception, trust, perceived attributes, and past experiences) which can act as potential factors to influence the belief of an individual. The integrated TPB model proposed in the current research (Figure 1), considered food safety and health consciousness as background variables. In fact, food safety and health consciousness are the behavioral determinants whose nature and importance in a migrated context may be relevant for all TPB constructs (Wu et al., 2016; Singh and Verma, 2017; Hansen et al., 2018), particularly in the current situation of COVID-19 (Olaimat et al., 2020; Qi et al., 2020).

In the migrated context, food safety and health concerns are major issues. Unhygienic food practices and consuming unfamiliar/local food can be a potential threat to the health of the sojourners (Wu et al., 2016; Yen et al., 2018). Mostly, not even after the consumption of unfamiliar local food, the sojourners can verify whether the food was hygienic, safe, and healthy (Xuhui et al., 2018). The food safety concerns in the migrated context can act as a potential barrier for sojourners to consume unfamiliar local and non-ethnic food. Consequently, in situations like COVID-19 outbreaks, it is important to thoroughly investigate the role of food safety and health consciousness with its strength to influence food consumption behavior.

Food safety describes the handling, processing, preparing, and storing the food in ways that help in preventing food-borne diseases (Gerard Fitzsimmons, 2012). The frequent food safety incidents and scandals in the Chinese food market, including the emergence of COVID-19, have increased the greater concerns of the sojourners with regard to food safety during their sojourning in China (Leone et al., 2020). During the emergence of COVID-19, the foodservice operators were among those who experienced the direct impact of the pandemic, which caused the potential source of infection (Aday and Aday, 2020). These types of concerns are consistent with food safety and are very important for food preferences. For instance, when the Avian flu (from birds to humans) erupted in Asia, the majority of Asian countries noticed a decrease in the tourists and in the consumption of poultry food (Chmielewski and Swayne, 2011).

Health consciousness is the perceived tendency to pay attention to one's health (Xu et al., 2020). More precisely, health consciousness is the self-awareness of an individual regarding his lifestyle, including seeking health information, natural environmental concerns, and food consumption (Hong, 2009). Several scholars argue that health concerns have a significant positive influence on the behavioral intentions of the consumers to consume healthy food (Hong, 2009; Singh and Verma, 2017; Qi et al., 2020). Hee and Jae-Eun (2011) identified health awareness as the most influential factor for food consumption behavior.

Some of the previous studies considered food safety and health consciousness as additional predictors of the behavioral intentions to purchase organic food when TPB was incorporated as a theoretical basis (Singh and Verma, 2017; Hansen et al., 2018; Ismael and Ploeger, 2020; Qi et al., 2020). Explaining the additional variables of intention variance within the classical TPB components, it was found that food safety and health consciousness are significant antecedents of intention. None of the studies have explored the extent to which relationships among food safety, health consciousness, and behavioral intentions were mediated by classical TPB components to avoid unfamiliar local food in the migrated context. Figure 1 summarizes the proposed mediated relationships between food safety, health consciousness, and consumption behavioral intentions of Pakistani students, where food safety and health consciousness are proposed as predictors of TPB components.

Previous studies found that food safety is an important predictor of the attitude of consumers when living abroad (Nevin and Ece, 2012; Lee et al., 2017). Employing TPB as a theoretical basis, food safety was recognized as an important precursor of attitude (Wu et al., 2016; Qi et al., 2020). Based on an extensive literature review, since the emergence of COVID-19, none of the studies in the field of the food preferences of sojourners have examined the associations among food safety, ATT, SN, and PBC. Few studies on the literature of tourism and hospitality (Maclaurin, 2004; Yeung and Yee, 2019) and on organic food consumption (Lobb et al., 2007; Alam et al., 2020; Qi et al., 2020) highlighted that food safety through attitude has a positive relationship with intentions and behavior. Capitalizing on the mentioned results concerning the link between food safety and attitude, we hypothesize that:

H4. Food safety has a significant positive effect on the attitude of Pakistani students to choose Pakistani cuisines over unfamiliar local food during their sojourning in China, particularly during the period of COVID-19.

According to Ajzen (2011), SN refers to the individual's perception of the belief of their important referents about a specific behavior. In our study, we argue that the positive association between food safety concerns and SN means that Pakistani students living in China and who have a higher level of food safety concerns will depend more on the beliefs of their referent. Confidence in important other beliefs plays an important role in determining the SNs (Canova et al., 2020). More precisely, we argue that China has a complex society, with the second-highest number of Pakistani international students who have a collectivistic cultural and religious identity, forms a social influence on each other. In fact, due to the increasing food safety concerns in the local Chinese food market, Pakistani students will comply more with the belief of their important others. Consequently, we hypothesize that:

H5. Food safety has a significant positive effect on the SN of Pakistani students to choose Pakistani cuisines over unfamiliar local food during their sojourning in China, particularly during the period of COVID-19.

Concerning PBC, food safety concerns in the local Chinese food market will motivate Pakistani students to gain control over consuming Pakistani cuisines and avoid unfamiliar local food products through self-efficacy. Self-efficacy is related to “an individual's perception of *being able* to perform the behavior.” Hence, we argue that the pure, safe, and healthy characteristics tethered to Pakistani cuisines dominate over the preferences for local food products and increase the self-efficacy of Pakistani students to consume only their cuisines. Based on this logic, we developed the following hypothesis:

H6. Food safety has a significant positive effect on the PBC of Pakistani students to choose Pakistani cuisines over unfamiliar local food during their sojourning in China, particularly during the period of COVID-19.

Similarly, previous researchers found health consciousness as an important predictor of consumer ATTs. Qi et al. (2020) investigated that during the COVID-19 pandemic, health consciousness is an influential factor of consumer attitude to consume green food. Singh and Verma (2017) found that health benefits have a significant positive impact on the attitude of Indian consumers to purchase organic food products. Similar deliberations were found in the study of Canova et al. (2020). With reference to ethnic food consumption, Ting et al. (2017) investigated that health considerations have a significant positive effect on the attitude of the Malaysian consumers to consume Dayak-food occasionally. Consequently, we argue that highly health conscious Pakistani students also prefer to avoid unfamiliar food products, particularly in the migrated context. Therefore, our next hypothesis is stated as follows:

H7. Health consciousness has a significant positive effect on the ATT of Pakistani students to choose Pakistani cuisines over unfamiliar local food during their sojourning in China, particularly during the period of COVID-19.

The key challenges these sojourners face in the host Chinese environment are the communication barrier, cultural and social disparities, and food consumption. The majority of the ethnic consumers of today are more interested in trying new foods but at the same time, they remain sensitive to their health benefits (Ma, 2015). In this situation, the motivation to comply with significant others is considered to be highly important to stay safe and healthy. The positive associations between health consciousness and SN mean that highly health conscious individuals will rely more on the belief of their important referent to avoid the possible risk associated with the consumption of unfamiliar local food. Therefore, our next hypothesis concerning the link between health consciousness and SN is as follows:

H8. Health consciousness has a significant positive effect on the SN of Pakistani students to choose Pakistani cuisines over unfamiliar local food during their sojourning in China, particularly during the period of COVID-19.

Health consciousness evaluates the readiness to perform healthy actions (Michaelidou and Hassan, 2008). Health conscious consumers are usually involved in maintaining their health and quality of life through healthy nutrition and physical fitness (Chmielewski and Swayne, 2011). Michaelidou and Hassan (2008) argued that health conscious consumers avoid unhealthy food; therefore, they have relatively higher control to opt for healthy and natural food. Similarly, health consciousness can act as a potential resource that will influence the control of Pakistani students to avoid unfamiliar local food during their sojourning in China. The positive attributes of Pakistani cuisines as being familiar, safe, and of better quality increase the control of Pakistani students to retain their cultural food and self-cooking, particularly during the crisis of COVID-19. Consequently, our next hypothesis is as follows:

H9. Health consciousness has a significant positive effect on the PBC of Pakistani students to choose Pakistani cuisines over unfamiliar local food during their sojourning in China, particularly during the period of COVID-19.

Finally, and as mentioned earlier, the three antecedents of behavioral intention (ATT, SN, and PBC) are assumed to mediate the structural relationships between food safety and consumption behavioral intention. Based on an extensive literature review, none of the studies on the area of food choices of the sojourners have analyzed that food safety and consumption behavioral intentions are mediated by classical TPB components. Some recent studies on organic food choices (Canova et al., 2020), the online service industry (Wu and Chen, 2005), and transport management (Madha et al., 2016) have analyzed that the classical TPB components mediate the relationships between consumer trust and intentions. Therefore, we proposed the following hypothesis.

H10a. Food safety has a positive and indirect relationship with behavioral intentions of Pakistani students to choose Pakistani cuisines over unfamiliar local food during their sojourning in China, through ATT.
H10b. Food safety has a positive and indirect relationship with behavioral intentions of Pakistani students to choose Pakistani cuisines over unfamiliar local food during their sojourning in China, through SN.
H10c. Food safety has a positive and indirect relationship with behavioral intentions of Pakistani students to choose Pakistani cuisines over unfamiliar local food during their sojourning in China, through PBC.

Similarly, some studies on the field of transport management (Hsiao and Yang, 2010), and (Madha et al., 2016) analyzed that the relationship between variety seeking and intentions to take high-speed rail are mediated *via* classical TPB components. As evident from the mentioned literature, we assume that the classical TPB components will also mediate the structural relationships between health consciousness and behavioral intentions of Pakistani students to retain the consumption of Pakistani cuisines and avoid unfamiliar local food in China. Consequently, we hypothesized as follows.

H11a. Health consciousness has a positive and indirect relationship with the behavioral intentions of Pakistani students to choose Pakistani cuisines over unfamiliar local food during their sojourning in China, through ATT.
H11b. Health consciousness has a positive and indirect relationship with the behavioral intentions of Pakistani students to choose Pakistani cuisines over unfamiliar local food during their sojourning in China, through SN.
H11c. Health consciousness has a positive and indirect relationship with the behavioral intentions of Pakistani students to choose Pakistani cuisines over unfamiliar local food during their sojourning in China, through PBC.

In conclusion, the current research aims to investigate the consumption behavioral intention of Pakistani students using the TPB model, along with extended measures of food safety and health consciousness, particularly during the COVID-19 crisis. Based on the available literature, we believe that our study is innovative for three reasons. First, the emergence of COVID-19 has considerably changed the global food production, processing, and consumption at different levels, but none of the studies, except the current research, has investigated the food preferences and consumption behavioral intention of sojourners during the COVID-19 crisis, particularly in China. Most of the studies (Butu et al., 2020; Laguna et al., 2020; Qi et al., 2020) have limited the investigations to organic and green food consumption during the crisis of COVID-19. Second, the role of food safety and health consciousness is examined, given its potential heuristic importance in the social-cognitive process occurring behind the creation and execution of behavior. Third, to the best of our knowledge, none of the studies on the field of food consumption have investigated that food safety and health consciousness have a positive and indirect relationship with behavioral intentions through the mediation of ATT, SN, and PBC. The results of our study will offer suggestions to different stakeholders concerned with the relationship between cognition and behavioral processes associated with food consumption, particularly in a migrated context.

## Materials and Methods

### Participants and Sampling Procedure

In this study, a quantitative approach was applied to examine the proposed model. All the data were collected with the help of organized, self-administered questionnaires distributed among Pakistani international students/Post-Doctoral Fellows registered as full-time students and living in different cities of China. The questionnaire was administered at the time when the pandemic situation was in a serious condition between July and August 2020. The respondents were those who were living in China since the COVID-19 outbreak and were highly concerned about the safety and health benefits of the food they consume. All the respondents signed a written consent and were briefed through an instruction document. Due to the lockdown and restrictions on free movement to combat the spread of coronavirus, a random sampling technique was applied online to directly approach all the respondents who took part in the study. Along with the consent letter, participants were provided with an instruction letter that informed them about the aim of the study. To ensure that the sample was not biased by a specific group of Pakistani students and to strictly follow the epidemic control and prevention policies, the questionnaire was spread *via* different social platforms (WeChat and QQ) of Pakistani international students through Questionnaire Star (online data collection software), where all the respondents have an equal chance of being selected. The targeted population completed 462 usable questionnaires, where all the respondents participated voluntarily.

The demographic profile of the respondents in the current study is mentioned in Table 1. The majority of the respondents were Pakistani students/Post-Doctoral Fellows who have registered in different disciplines and who have spent 2–5 years in China. Considering the Covid-19 pandemic facts, health consciousness, communication barrier, and cultural and social disparities, the respondents of the current study were expected to be less assimilated to the host Chinese culture. Regarding the gender distribution of collected survey data, it was found that 57.35% of the respondents were men, whereas 42.64% were women. With regard to the age of the respondents, it was found that 38.74% were under the age of 25, while 54.73% were aged 26–35 years, and 5.89% and 0.64% were between 36–45 and 46–55 years, respectively. A total of 23.16% were undergraduate students, whereas 76.82% were postgraduate or Ph.D. students.

**Table 1:** Characteristics of survey sampling (*n* = 462).

**Demographics**		**Statistics**	
	**Specifications**	***N***	**%**
Gender	Male	265	57.35
	Female	197	42.64
Age	Under-25	179	38.74
	25–35	253	54.73
	36–45	27	5.89
	45–55	3	0.64
Qualification	Undergraduate	107	23.16
	Master	223	48.26
	PhD	129	27.92
	Others	3	0.64

### Measures

The measures of the TPB construct adopted in the current research were those used by previous researchers in different contexts, which comply with the guidelines for the construction of the TPB questionnaire. All the classical TPB model constructs along with the background constructs shown in Figure 1 were regarded as latent variables. The questionnaire used for the data collection comprised of two portions. The demographic information of the participants was placed in the first part. In the second part of the questionnaire, the food consumption behavioral intention of the targeted population during Covid-19 based on the classical TPB components was measured. The ATT of the participants was evaluated through four items derived from the proposed scale by Qi et al. (2020) and Alam et al. (2020), whereas the adoption of five items from the proposed scale by Ali et al. (2018) and Singh and Verma (2017) measured the SN. The PBC was measured by seven items adapted from Bonne et al. (2007) and Ting et al. (2016). The consumption behavioral intention (CBI) to consume Pakistani food was measured with the help of six items proposed by Yen et al. (2018) and Qasim et al. (2019). The food safety (FS) concern of the consumers was measured by four items derived from Wu et al. (2016) and Qi et al. (2020). Four items from the study of Hong (2009) and Hansen et al. (2018) were used to measure the health consciousness (HC). All the items were measured through a seven-point Likert Scale ranging from “strongly disagree” (1) to “strongly agree” (7). To ensure the response format and reliability of the responses of the sojourners, the questionnaire was piloted with 50 respondents.

### Common Method Variance

The current research contributes to the data obtained from the same participants for both variables (independent and dependent). Therefore, there is a possibility for the existence of method biasness. To reduce method biasness, our study ensures that all the participants were briefed properly; this helped them to completely understand the questionnaire. The main approaches adopted for this reason include the variance inflation factor (VIF) and tolerance (TOL) test. The results shown in **Table 4** suggest that the TOL values are >0.1, and the observed values of the VIF are <10, indicating no collinearity issue in the study.

### Model Assessment

The proposed relationships of the current research model were converted into structural equation modeling (SEM) for a further analysis comprising an outer model and an inner model. The PLS-SEM was used *via* Smart PLS 3. This study opted for PLS-SEM as an appropriate multivariate technique, which is used for understanding the relatively complex models and the multivariate relationships among them. In the field of management, the PLS-SEM has relished its attractiveness as a useful multivariate analytical technique. The flexibility and adequacy of the model have been recognized by the strategic management research to analyze multiple relationships among variables (Sarstedt et al., 2014).

## Results

### Partial Least Square Structural Equation Modeling

The study used a two-step approach to evaluate the proposed research model. Initially, the outer model (measurement model) assessment was carried out, which evaluated the validity and reliability of the scale adopted in the study. The next step assessed the inner model (structural model) to measure the fitness of the model and the proposed relationships among variables. For this purpose, the PLS-SEM version 3 was used.

### Measurement Model

For the evaluation of the reflective measurement model, the convergent and discriminant validity assessment was considered. Tables 2, 3 depict the specific results of the assessment. Convergent validity states that the items which measure the same construct should be highly correlated (Hair et al., 2019). It was assessed through factor loading and composite reliability along with the average variance extracted (AVE). The factor loadings for all the items ranged from 0.621 to 0.876 (above 0.5), which are satisfactory. The values of the composite reliability (CR) also ranged from 0.842 to 0.928, which are greater than the required range (0.7), thus demonstrating a good proportion of internal consistency. Finally, the AVE for the item loading was examined. The AVE is calculated by adding all the squared factor loadings of items in the construct divided by their numbers. The results demonstrated that the AVE for all of the constructs ranged from 0.548 to 0.721, which is higher than the minimum threshold value of 0.5, thus ensuring that all the items explain a variance of more than 50% in the constructs (Hair et al., 2017).

**Table 2:** Convergent validity assessment (*n* = 462).

**Constructs and items**	**Items**	**CL**	**CR**	**AVE**
**Attitude**				
The Pakistani food is important to me.	ATT1	0.82	0.90	0.70
Consuming Pakistani food is a reasonable action for me.	ATT2	0.84		
Eating Pakistani food is a positive activity for me.	ATT3	0.82		
I feel worried if Pakistani food is not available to me.	ATT4	0.78		
**Subjective norm**				
My close friends/family consume our ethnic food.	SN1	0.62	0.86	0.54
My loved ones expect me to consume my ethnic food only.	SN2	0.83		
People who are important to me think I should eat my food.	SN3	0.84		
My referents pressurize me to consume Pakistani food only.	SN4	0.64		
The institutions I follow encourage me to eat Pakistani food.	SN5	0.74		
**Perceived behavioral control**				
I have enough control over eating Pakistani food.	PBC1	0.84	0.84	0.62
Pakistani food is easily available in the market.	PBC2	0.87		
I can buy Pakistani food online.	PBC3	0.62		
**Food safety**				
The pandemic has increased my food safety concerns.	FS1	0.86	0.90	0.70
I am concerned about the safety of the local Chinese food.	FS2	0.82		
I am convinced about the safety of Pakistani food.	FS3	0.85		
I am being panicked by food safety issues during the pandemic.	FS4	0.81		
**Health consciousness**				
The pandemic has hyped the level of my health consciousness.	HC1	0.86	0.89	0.67
I carefully choose food to ensure good health.	HC2	0.84		
I believe that Pakistani food is healthy.	HC3	0.80		
I am very concerned about my health during the pandemic.	HC4	0.77		
**Consumption behavioral intentions**				
I always prefer to eat Pakistani food.	CBI1	0.85	0.92	0.72
I am always more interested in consuming Pakistani food.	CBI2	0.87		
I intend to consume Pakistani food in the future.	CBI3	0.82		
I will make special efforts to consume Pakistani food.	CBI4	0.84		
I would choose to eat Pakistani food even if it costs more.	CBI5	0.84		
Given a choice between two, I intend to choose Pakistani food.	CBI6	0.82		

*CL, cross loadings; CR, composite reliability; AVE, average variance extracted; ATT, attitude; SN, subjective norm; PBC, perceived behavioral control; CBI, consumption behavioral intentions; FS, food safety; HC, health consciousness*.

**Table 3:** Discriminant validity assessment (*n* = 462).

**Hetrotrait-Monotrait Ratio (HTMT)**
Attitude						
Consumption behavioral intention	0.839					
Food safety	0.779	0.795				
Health consciousness	0.773	805	0.743			
Perceived behavioral control	0.498	0.428	0.539	0.519		
Subjective norm	0.82	0.772	0.745	0.731	0.456	

*Source: Estimated results based on Henseler et al. (2015) heterotrait-monotrait (HTMT) criterion*.

Discriminant validity was assessed through the criterion, Henseler's heterotrait-monotrait (HTMT) (Henseler et al., 2015). The results of HTMT are depicted in Table 3. Following the suggestions of Henseler et al. (2015), the obtained values of HTMT ratios mentioned in Table 3 were lower than 0.85. Therefore, the results of the discriminant validity for each of the constructs in the proposed model are satisfactory.

### Structural Model Assessment

Before the structural model assessment, it is necessary to make sure that the structural model does not pose any problems of collinearity. The result of the collinearity assessment is presented in Table 4, which depicts that in each of the constructs, the values of VIF are smaller than the offending value of 10, whereas the TOL values are >0.1, which indicates no multicollinearity issues in the current study.

**Table 4:** Collinearity assessment.

**IVs**	**Tolerance**	**VIF**
ATT	0.410	2.591
FS	0.523	2.094
HC	0.539	2.081
PBC	0.619	1.402
SN	0.449	2.29

*IVs, independent variables; ATT, attitude; SN, subjective norm; PBC, perceived behavioral control; FS, food safety; HC, health consciousness; VIF, variance inflation factor.*

*Source: Estimated results based on the collinearity assessment by Latan and Noonan (2017)*.

**Figure 2:**
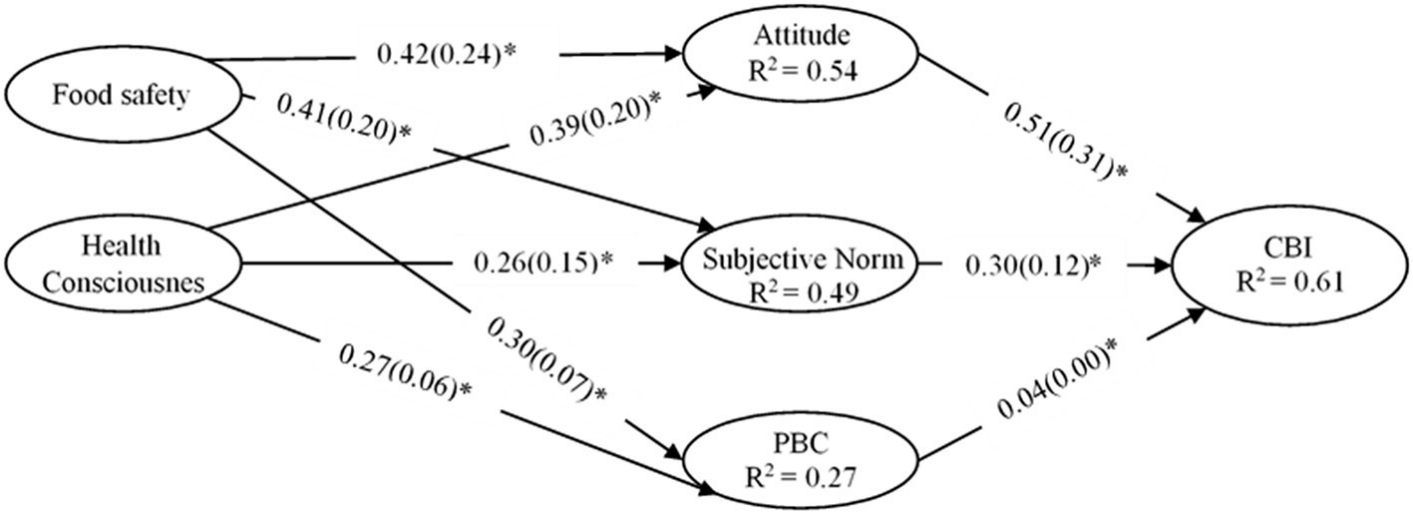
Structural equation modeling **(**SEM) results of complete data (*n* = 462), PBC, Perceived Behavioral Control. The * indicates *p*-values <0.01. The figure presents the effect sizes (*f*^2^) in the parentheses next to each path coefficient (β).

For the assessment of the proposed structural model (Figure 2), the dimensions and values of standardized path coefficients with other related *t*-statistics, including the calculation of *R*^2^ (coefficient of determination) were considered important. The study applied the resampling technique (i.e., bootstrapping) to 5,000 resamples for measuring the path coefficients and their relative importance in the proposed model. The study also considered measuring the effect sizes (*f*^2^) for the proposed structured paths as suggested by Hair et al. (2017). Moreover, to measure the predictive ability of the proposed model, Stone–Geisser's Q^2^ was also taken into consideration.

The results of β-coefficients, t-values, and *f*^2^-values for each of the respective structural paths obtained from the bootstrapping procedure are illustrated in Table 5. All of the proposed relationships, except one, were found to be significant at the confidence level of 99.0 percent. Attitude (ATT → CBI, β = 0.615, *t* = 11.053, LL = 0.417, UL = 0.609, *P* ≤ 0.01) showed the strongest positive effect on the consumption behavioral intentions of Pakistani international students to retain Pakistani cuisines when living in China. Subjective norm (SN → CBI, β = 0.319, *t* = 6.648, LL = 0.219, UL = 0.412, *P* ≤ *0.01*) was also positively associated with the consumption behavioral intention. Hence, H1 and H2 were supported by the results. However, PBC (PBC → CBI, β = 0.040, *t* = 0.978, LL = −0.037, UL = 0.125, *P* ≥ *0.05*) does not affect the consumption behavioral intentions to retain Pakistani cuisines, thus rejecting H3. With regard to background factors, food safety concern was positively associated with ATT (FS → ATT, β = 0.427, *t* = 9.421, LL = 0.337, UL = 0.514, *P* ≤ *0.01*), PBC (FS → PBC, β = 0.306, *t* = 5.147, LL = 0.196, UL = 0.410, *P* ≤ *0.01*), and perceived social pressure (FS → SN, β = 0.414, *t* = 8.782, LL = 0.317, UL = 0.498, *P* ≤ *0.01*), that sustained H4, H5, and H6, respectively. Similarly, the results revealed that health consciousness was positively associated with ATT (HC → ATT, β = 0.393, *t* = 8.469, LL = 0.299, UL = 0.490, *P* ≤ *0.01*), PBC (HC → PBC, β = 0.273, *t* = 4.993, LL = 0.168, UL = 0.380, *P* ≤ *0.01*), and perceived social pressure (HC → SN, β = 0.363, *t* = 7.674, LL = 0.281, UL = 0.445, *P* ≤ *0.01*) as expected from H7, H8, and H9.

**Table 5:** Assessment of structural paths (Hypothesis testing).

**Structural paths**	**β-Value**	**t-value**	***f*^2^**	**LL**	**UL**	**Results**
ATT → CBI	0.515	11.053^**^	0.314	0.417	0.609	Supported
PBC → CBI	0.040	0.978	0.003	−0.037	0.125	Not supported
SN → CBI	0.309	6.648^**^	0.123	0.219	0.412	Supported
FS → ATT	0.427	9.421^**^	0.242	0.337	0.514	Supported
FS → SN	0.414	8.782^**^	0.203	0.317	0.498	Supported
FS → PBC	0.306	5.147^**^	0.078	0.196	0.410	Supported
HC → ATT	0.393	8.469^**^	0.206	0.299	0.490	Supported
HC → SN	0.363	7.674^**^	0.156	0.281	0.445	Supported
HC → PBC	0.273	4.993^**^	0.062	0.168	0.380	Supported

** *Significance at p ≤ 0.01.*

*In the current study, the intentions were to retain Pakistani food consumption during COVID-19. Pakistani students have positive intentions toward consuming their own food when living in China.*

*ATT, attitude; SN, subjective norm; PBC, perceived behavioral control; CBI, consumption behavioral intentions; FS, food safety; HC, health consciousness. LL, lower limit; UL, upper limit at 99 percent confidence interval*.

The Cohen (Cohen, 1970) criteria of (0.02) for small, (0.15) for medium, and (0.35) for large-size effects was adapted to measure the effect sizes (*f*^2^). All of the variables, except one, exceeded the minimum threshold criterion of (0.02), thus reflecting its effect on the dependent variable (small-to-medium size). However, the PBC (PBC, *f*^2^ = 0.003) exhibited no considerable effect on the consumption behavioral intention. Table 5 illustrates the overall (*f*^2^) results.

Besides, we also evaluated the coefficient of determination (*R*^2^) along with the predictive relevance (Q^2^) of independent variables on dependent variables. The computed value of *R*^2^ for the dependent variable (CBI) was (0.616), which indicates that the overall independent variables (ATT, SN, and PBC) in the present study explain (61.6%) variance in the dependent variable (CBI). Similarly, for background factors, the *R*^2^ accounted for dependent variables (ATT, SN, and PBC) were (0.546), (0.490), and (0.271), respectively. This result indicates that food safety and health consciousness explain 54.6, 49, and 27.1% of variances in the dependent variables of ATT, SN, and PBC, respectively.

In addition, adopting the procedures recommended by Shmueli et al. (2019). The PLS predict was also performed. In order to assess the predictive validity, the cross-validation with holdout sampling technique was applied. The overall assessment results are depicted in Table 6. Initially, the Q^2^ (the comparison of PLS path model and the simple mean prediction) values were measured. The respective Q^2^ values of (0.666), (0.541), (0.485), and (0.256) for CBI, ATT, SN, and PBC suggest the appropriate predictive performance of the proposed model. Secondly, to generate predictions, the linear regression model (LM) was followed as suggested by Shmueli et al. (2019). The results suggest that comparing the LM and PLS results, the LM outcomes have lower prediction error in terms of root mean square error (RMSE) and mean absolute error (MAE), indicating the substantial predictive power of the model.

**Table 6:** PLS predict assessment.

**PLS prediction summary**									
									
CBI		0.666							
ATT		0.541							
SN		0.485							
PBC		0.256							
**PLS prediction summary**									
	**PLS**			**LM**			**PLS-LM**		
	**RMSE**	**MAE**	**Q** ^**2**^ **Predict**	**RMSE**	**MAE**	**Q** ^**2**^ **Predict**	**RMSE**	**MAE**	**Q** ^**2**^ **Predict**
ATT2	1.287	0.908	0.342	1.37	0.962	0.317	−0.083	−0.054	0.025
ATT1	1.385	0.962	0.301	1.255	0.875	0.374	0.13	0.087	−0.073
ATT3	1.222	0.907	0.455	1.232	0.915	0.446	−0.01	−0.008	0.009
ATT4	1.447	1.137	0.404	1.448	1.113	0.404	−0.001	0.024	0
CBI4	1.134	0.871	0.489	1.215	0.865	0.484	−0.081	0.006	0.005
CBI6	1.125	0.837	0.427	1.156	0.83	0.529	−0.031	0.007	−0.102
CBI5	1.172	0.929	0.522	1.016	0.728	0.539	0.156	0.201	−0.017
CBI2	1.19	0.897	0.501	1.079	0.777	0.538	0.111	0.12	−0.037
CBI3	1.078	0.804	0.482	1.037	0.752	0.626	0.041	0.052	−0.144
CBI1	1.25	0.946	0.455	1.067	0.781	0.485	0.183	0.165	−0.03
PBC6	1.748	1.497	0.027	1.389	1.023	0.228	0.359	0.474	−0.201
PBC7	1.715	1.449	0.019	1.406	1.037	0.183	0.309	0.412	−0.164
PBC2	1.422	1.063	0.163	1.615	1.217	0.067	−0.193	−0.154	0.096
PBC5	1.747	1.455	0.072	1.794	1.518	0.056	−0.047	−0.063	0.016
PBC1	1.392	1.04	0.225	1.777	1.476	0.04	−0.385	−0.436	0.185
PBC4	1.822	1.568	0.026	1.754	1.494	0.019	0.068	0.074	0.007
PBC3	1.638	1.252	0.039	1.713	1.432	0.02	−0.075	−0.18	0.019
SN5	1.602	1.288	0.215	1.282	0.923	0.293	0.32	0.365	−0.078
SN3	1.454	1.163	0.393	1.553	1.265	0.347	−0.099	−0.102	0.046
SN1	1.289	0.938	0.285	1.466	1.154	0.383	−0.177	−0.216	−0.098
SN4	1.788	1.47	0.056	1.766	1.467	0.08	0.022	0.003	−0.024
SN2	1.561	1.292	0.34	1.604	1.305	0.213	−0.043	−0.013	0.127

*ATT, attitude; SN, subjective norm; PBC, perceived behavioral control; CBI, consumption behavioral intentions; FS, food safety; HC, health consciousness; LM, linear regression model; RMSE, root mean square error; MAE, mean absolute error*.

### Mediation Effect of TPB Components Among Background Factors and Behavioral Intention

Our theoretical model proposed that TPB components will mediate the structural relationships between background factors (food safety and health consciousness) and the consumption behavioral intentions of Pakistani students to retain the consumption of Pakistani cuisines during their sojourning in China, particularly during the period of COVID-19 (H10a,b,c and H11a,b,c). To examine the mediation effect, and as suggested by Hair et al. (2019), the resampling technique of bootstrapping to generate 5,000 resamples was followed. To report the outcomes generated from each mediation path in the model, we then employed the function of the specific indirect-effect in Smart-PLS (Hair et al., 2019). The *P*-values, along with the 95% confidence intervals (bias-corrected), are also reported in Table 7 to ensure the significant importance of the proposed indirect results. The results from the PLS-SEM function of the specific indirect effect revealed that the indirect effect of food safety on the consumption behavioral intention through the mediation of ATT (β = 0.22, LL = 0.159, UL = 0.272, *P* ≤ *0.00*) and SN (β = 0.128, LL = 0.081, UL = 0.172, *P* ≤ 0.00) was significant. Similarly, the indirect effect of health consciousness on the consumption behavioral intention through the mediation of ATT (β = 0.203, LL = 0.138, UL = 0.279, *P* ≤ *0.00*) and SN (β = 0.112, LL = 0.069, UL = 0.163, *P* ≤ *0.00*) turned out to be significant. However, the specific indirect effect of food safety and health consciousness on the consumption behavioral intention *via* PBC (β = 0.012, LL = −0.013, UL = 0.040, *P* ≤ *0.336*) and (β = 0.011, LL = −0.009, UL = 0.043, *P* ≤ *0.412*) turned out to be insignificant. Therefore, based on the results, we concluded that the effect of food safety and health consciousness on CBI was mediated by ATT and SN, whereas the PBC does not mediate this relationship.

**Table 7:** Mediation effect.

**Structural paths**	**β-Value**	**t-Value**	***P*-values**	**LL**	**UL**	**Status**
FS → ATT → CBI	0.220	7.303	0.000^**^	0.159	0.272	Supported
FS → PBC → CBI	0.012	0.963	0.336	−0.013	0.040	Not supported
FS → SN → CBI	0.128	5.441	0.000^**^	0.081	0.172	Supported
HC → ATT → CBI	0.203	5.678	0.000^**^	0.138	0.279	Supported
HC → PBC → CBI	0.011	0.822	0.412	−0.009	0.043	Not supported
HC → SN → CBI	0.112	4.400	0.000^**^	0.069	0.163	Supported

** *Significance at p ≤ 0.01.*

*ATT, attitude; SN, subjective norm; PBC, perceived behavioral control; CBI, consumption behavioral intentions; FS, food safety; HC, health consciousness; LL, lower limit; UL, upper limit at 99% confidence interval*.

## Discussion and Conclusion

The emergence of COVID-19 has placed a revolutionary strain on the global food system, which has caused changes in the way food is prepared, sold, obtained, and consumed (Leone et al., 2020). With these uncertainties, the sustainable food consumption is perceived as a major issue, particularly in a migrated context (Alam et al., 2020). The frequent food safety incidents and scandals in the Chinese food market, including the emergence of COVID-19, have increased greater concerns of the sojourners with regard to food safety and health consciousness (Qi et al., 2020). In contrast, none of the research has investigated the consumption patterns of the sojourners during the COVID-19 pandemic, particularly in Mainland China. The current research contributes to an appreciation of the reasons for the reluctance of young Pakistani students living in China to consume unfamiliar local food products available in the Chinese food market while preferring to retain the consumption of Pakistani cuisines during the period of the COVID-19 outbreak. In particular, the current research follows one of the highly significant socio–psychological theoretical frameworks, TPB, along with the background factors of food safety and health consciousness.

The findings of the current research highlighted that the TPB is a valuable framework for understanding the desired investigated behavior and has a strong explanatory power. More precisely, the relationships among ATT, SN, and consumption behavioral intentions were found to be significant, which supported H1 and H2; however, the relationship between PBC and consumption behavioral intention (H3) did not receive any support.

Firstly, through investigating the direct relation between TPB components, we found that a positive attitude of young Pakistani students toward Pakistani cuisines predict intention, and indirectly the actual behavior. Consequently, avoiding unfamiliar local food and retaining the consumption of Pakistani cuisines while living in China, supporting H1. The results also confirmed that the consumption behavioral intentions are strongly supported by attitude. This result further suggests that Pakistani students have a more promising evaluation of consuming Pakistani cuisines with a greater likelihood of being involved in actual consumption behavior. Our results are consistent with the previously published literature of Ali et al. (2017) and Sadia et al. (2021); these researchers found a positive relationship between attitude and behavioral intentions of Muslim students to consume halal when living abroad.

Secondly, SN also affects the consumption behavioral intentions of Pakistani students living in China, strongly supporting H2. The SN plays the role of the second most significant determinant of intention. The positive impact of SN reflects that the discussions on the current food scandals and safety concerns in the Chinese food market with referents, such as close friends and family members, positively affect their willingness to avoid unfamiliar food products while living in China. Expectations about food consumption shared with important referent and their effect on the food choices of the consumers are reported in the recent work of Canova et al. (2020) in the field of organic food industry.

Our results did not provide support for H3, suggesting that PBC is not associated with the consumption behavioral intentions of Pakistani students to consume Pakistani cuisines during their stay in China. This might be due to the perceived lack of availability and relatively high cost of Pakistani food ingredients in the local Chinese food market, which becomes the potential barrier for these sojourners maintaining their eating habits. Moreover, the epidemic prevention and control policies and lockdown to combat the spread of the virus is also an obstacle for the behavioral control. Our results are confirmatory to the findings of Verbeke and López (2005), who found PBC as a potential obstacle for Hispanic consumers preserving their eating habits while living in Belgium. In contrast, some scholars contended that the perceived lack of availability or a relatively high cost of food ingredients would not affect the desired consumption behavior if the importance or personal relevance attached to that food dominates With regard to the background factors, H4 received support since food safety was positively related to attitude, thus mirroring the existent literature (Nevin and Ece, 2012; Wu et al., 2016; Lee et al., 2017). These researchers found a positive effect of food safety on attitude to handle on-campus food practices of university students. Moreover, food safety explained a higher quota of the ATT variance. The results also highlighted that food safety has positive associations with SN and PBC, resultantly supporting H5 and H6. Besides, food safety explained a relatively lower quota of PBC variance, as compared to ATT and SN. Similarly, H7, H8, and H9 received support based on the results. This suggests that health consciousness had a significant positive effect on the attitude of the sojourners, which reflects the extant literature available on organic food (Singh and Verma, 2017; Hansen et al., 2018; Qi et al., 2020). Similar to food safety, health consciousness also had positive associations with SN and PBC.

Based on these results, we argue that food safety and health consciousness play a vital role in the development of an overall positive evaluation of Pakistani students toward retaining their ethnic food consumption during their sojourning in China. The positive effect of food safety and health consciousness on attitude reveals that the more the sojourners are health conscious and trustworthy with regard to the safety aspects of their ethnic food, the more they will have a positive attitude toward it. Similarly, the positive influence of food safety and health consciousness on SN articulated the higher safety concerns regarding unfamiliar local food. To this extent, the Pakistani students living in China believe themselves to be more vulnerable to possible food-borne diseases; the more they will be health conscious and follow the opinions of important others. Subsequently, the positive effect of food safety and health consciousness on PBC suggests that the health and safety concerns associated with consuming unfamiliar local Chinese food facilitate Pakistani students to devote more efforts to retain their ethnic food consumption.

Our results also concluded that with increasing health and safety concerns in the local food market, eating “home food” remains the main focus of Pakistani students living in China. Eating “home food” involves self-cooking at home/student dormitories since Pakistani restaurants and take-away are too expensive and less authentic for being considered as healthy routine options. The observed behavior of the Pakistani students are in line with the results of Kamran (2021), where the respondents reported that the taste and spices of Chinese local food are different from their ethnic cuisines. Besides, self-cooking within the accommodations of the students and sharing a Pakistani meal with fellow Pakistani students strengthen the bond between them, thus helping them to maintain their cultural norms and consumption habits. Cooking and eating Pakistani food together becomes a part of the SN, which helps them in coping strategies to navigate the possible uncertainties and vulnerabilities associated with eating unfamiliar local food in a new environment. With regard to PBC, the positive association of food safety and health consciousness with behavioral control means that the acquisition of Pakistani food ingredients in the local Chinese food market does not remain a problem for Pakistani students. They have learned how and where to buy food ingredients. Even if the supply of the required ingredients in some locations is limited, these sojourners can learn to improvise the original recipes with available new ingredients through the inter-cultural adaption process. For example, the inter-cultural adaption process of the Chinese international students in Europe to replace the original Chinese Lamian noodles with spaghettis, fried with some pickled vegetables and pork meat is the best example available in the literature (Yen et al., 2018). However, the actual behavioral control of Pakistani students to consume their desired food is subject to the pandemic situation and the supply of food ingredients in the local Chinese food market.

Finally, we checked whether TPB classical components lead to mediation between background factors (food safety and health consciousness) and consumption behavioral intentions. The results revealed that food safety and health consciousness were indirectly associated only with behavioral intentions through their substantial effects on ATT (H10a, H11a) and SN (H10b, H11b). The results did not provide support for the indirect association between food safety, health consciousness, and behavioral intention *via* PBC (H10c and H11c). Thus, the overall mediation hypothesis (H10a,b,c and H11a,b,c) were partially supported by the results. The reason for this result might be due to the inverse effects of COVID-19 on movement restrictions, closure of production facilities, and food trade restrictions that caused the perceived lack of availability and the relatively high cost of Pakistani food ingredients in the Chinese food market.

The proposed model, which incorporated food safety and health consciousness as background variables in the classical TPB framework, produced vigorous results to explain the consumption behavioral intention of Pakistani students living in China. However, the background factors were added to assess the role of food safety and health concerns of Pakistani international students, regarding their food preferences during the period of COVID-19. We are aware that this is an operationalization within the classical framework of TPB, which has never been validated nor has been applied to investigate the consumption patterns of the academic sojourners in the previous framework. Nevertheless, within the context of the current study, the introduction of food safety and health consciousness within the framework of TPB was deemed important to assess the presumed determination of intentions to avoid unfamiliar food products during the COVID-19 pandemic. Hence, the background factors have been proved to be acting as additional predictors within the TPB model to investigate the desired behavior.

## Practical Implications

With regard to the practical implications, the findings of the current research are useful for different stakeholders, including food producers, marketers, policymakers, and even businesses attracted by the ethnic food sector. Firstly, the current study revealed that in response to the health and safety concerns in the wake of COVID-19 pandemic, Pakistani students living in China desire to strive for a safe and healthy life, allocate time, and efforts to prepare their food. Therefore, the Chinese food producers and suppliers need to accommodate it as a market demand. They need to improve the demand-based imports and supply of Pakistani food ingredients and establish effective supply chains to ensure their availability to the consumers.

Secondly, due to the epidemic control and prevention policies implemented by the Chinese government, the PBC of Pakistani international students living in China was adversely affected to retain their ethnic food, thereby, hampering the availability and affordability of Pakistani food ingredients to prepare and consume their ethnic food. The public policies regarding uninterrupted global trade, removing restrictions, and maintaining food and feed supplies need to be revived to enable these sojourners to regain their consumption behavior impeded by COVID-19 control measures.

Thirdly, based on the health and safety concerns identified by this research, improvements could be made to the food provided on the university campus and in the area populated by Pakistan international students. Access to the Chinese Muslim food known as Qing Zhen (clean) might help Pakistani students to overcome their concerns about the reliability of local food as well as to offset their anxiety to avoid any kind of food-borne diseases.

Finally, the findings of the current study are generally applicable for other food sectors, particularly during the period of COVID-19. With the emergence of COVID-19, majority of the consumers today prefer to consume healthy food products. Therefore, food producers and marketers of other food sectors need to emphasize on the quality and safety of their products. Moreover, this study also contributes to formalize restaurants and food cafes to ensure food safety and quality standards.

The current research also contributes to the extant literature on the food consumption of the sojourners in different ways. Firstly, it has contributed to the scarce research on the food consumption of Pakistani international students in terms of health and safety concerns during the COVID-19 pandemic. Secondly, the antecedents of the decision of the sojourners to consume their ethnic food in the wake of COVID-19 in a migrated context, adopting a notorious socio-psychological theory, that is, the TPB, were thoroughly investigated. Thirdly, it presented the associations between food safety, health consciousness, and consumption behavioral intention through the mediation of classical TPB components, which are rarely investigated.

Despite its strength, there are few caveats related to the current research. Firstly, due to the use of convenience sampling, generalizability of the present study to entire Pakistani academic sojourners is questionable. Secondly, due to the epidemic control and prevention policies in China, our study is limited to the consumption behavioral intentions phase. The actual behavior of the target population will be assessed in the second phase of the study once the epidemic control and prevention policies are relaxed. Finally, the examination of some additional factors, such as dietary acculturation, consumption values, and consumer trust can also be accompanied by relationships considered in the current research.

To fully understand the role of food safety and health consciousness and their antecedents in the food consumption process, particularly in an unfamiliar food market, future researchers can utilize the current extension in the TPB model to predict food consumption behavior in other food sectors as well.

## Data Availability Statement

The raw data supporting the conclusions of this article will be made available by the authors, without undue reservation.

## Ethics Statement

The studies involving human participants were reviewed and approved by the research ethics committee at China University of Geosciences (Wuhan), China. The patients/participants provided their written informed consent to participate in this study.

## Author Contributions

MK and SC conceived and designed the study. MK, NK, and AT collected the data. MK and HQ developed the theoretical framework. MK and LJ performed the data analysis. SC and HQ verified the analytical methods. MK wrote the first draft. SC, MI, and RA substantially revised the manuscript. All authors discussed the results and contributed to the final manuscript.

## Conflict of Interest

The authors declare that the research was conducted in the absence of any commercial or financial relationships that could be construed as a potential conflict of interest.

## Publisher's Note

All claims expressed in this article are solely those of the authors and do not necessarily represent those of their affiliated organizations, or those of the publisher, the editors and the reviewers. Any product that may be evaluated in this article, or claim that may be made by its manufacturer, is not guaranteed or endorsed by the publisher.
